# Assessment of Inflammatory Hematological Ratios (NLR, PLR, MLR, LMR and Monocyte/HDL–Cholesterol Ratio) in Acute Myocardial Infarction and Particularities in Young Patients

**DOI:** 10.3390/ijms241814378

**Published:** 2023-09-21

**Authors:** Bogdan-Sorin Tudurachi, Larisa Anghel, Andreea Tudurachi, Radu Andy Sascău, Cristian Stătescu

**Affiliations:** 1 Internal Medicine Department, “Grigore T. Popa” University of Medicine and Pharmacy, 700503 Iași, Romania; bogdan-sorin.tudurachi@d.umfiasi.ro (B.-S.T.); radu.sascau@umfiasi.ro (R.A.S.); cristian.statescu@umfiasi.ro (C.S.); 2 Cardiology Department, Cardiovascular Diseases Institute “Prof. Dr. George I. M. Georgescu”, 700503 Iași, Romania; leonteandreea32@gmail.com

**Keywords:** acute myocardial infarction, neutrophil/lymphocyte ratio, platelet/lymphocyte ratio, monocyte/lymphocyte ratio, lymphocyte/monocyte ratio, young patients

## Abstract

Cardiovascular disease, particularly coronary artery disease (CAD), remains a predominant cause of mortality globally. Factors such as atherosclerosis and inflammation play significant roles in the pathogenesis of CAD. The nexus between inflammation and CAD is underscored by the role of immune cells, such as neutrophils, lymphocytes, monocytes, and macrophages. These cells orchestrate the inflammatory process, a core component in the initiation and progression of atherosclerosis. The activation of these pathways and the subsequent lipid, fibrous element, and calcification accumulation can result in vessel narrowing. Hematological parameters derived from routine blood tests offer insights into the underlying inflammatory state. Recent studies have highlighted the potential of inflammatory hematological ratios, such as the neutrophil/lymphocyte ratio, platelet/lymphocyte ratio, monocyte/lymphocyte ratio and lymphocyte/monocyte ratio. These parameters are not only accessible and cost-effective but also mirror the degree of systemic inflammation. Several studies have indicated a correlation between these markers and the severity, prognosis, and presence of CAD. Despite the burgeoning interest in the relationship between inflammatory markers and CAD, there remains a paucity of data exploring these parameters in young patients with acute myocardial infarction. Such data could offer valuable insights into the unique pathophysiology of early-onset CAD and improve risk assessment and predictive strategies.

## 1. Introduction

Cardiovascular disease is still the leading cause of death worldwide, despite all the therapeutic advances. According to the American Heart Association statistics, in the United States, one person dies every 34 s from cardiovascular disease, and one in every five deaths in 2020 were caused by heart disease [1]. Unfortunately, coronary artery disease is the most common type of heart disease, being encountered in 7.2% of adults over 20 years old. Statistics show that, in 2020, about 2 in 10 deaths from coronary artery disease happened in adults younger than 65 years old [2]. The main cardiovascular risk factor for coronary artery disease is atherosclerosis. Inflammation has an important role in the initiation and progression of the atherosclerotic process [3]. The activation of inflammatory pathways, and also endothelium activation, with the accumulation of lipids, fibrous elements and calcification, will lead to vessel narrowing [4]. The complex interaction between the cells of the immune system, such as neutrophils, lymphocytes, monocytes and macrophages, contributes to the appearance of inflammation. Thus, different studies have demonstrated that the presence and degree of chronic inflammation is reflected by the number and percentage of immune-system-related cells [5]. Generally, hematological parameters measured in routine blood tests mainly include quantifying and classifying the white and red blood cells, and also platelets. Recent studies have shown that red blood cells distribution width (RDW), mean platelet volume (MPV), the neutrophil/lymphocyte ratio (NLR, neutrophil count/lymphocyte count), platelet/lymphocyte ratio (PLR, platelet count/lymphocyte count) and monocyte/lymphocyte ratio (MLR, monocyte count/lymphocyte count) are accessible and cost-effective inflammatory markers that reflect the degree of inflammation [5,6,7]. It is known that coronary artery disease is characterized by a high inflammatory burden, and several studies demonstrated that these hematological parameters are associated with the severity and prognosis of coronary artery disease [8,9]. Another important modification that can be found in patients with CAD is the change in lipid profile tests. Modern lifestyles increased the number of young patients suffering from coronary artery disease. Thus, in recent times, we observed that CAD occurs frequently in individuals younger than 50 years of age, which is considered as early-onset coronary artery disease. The etiology and also the risk factors are different between early- and late-onset CAD [10]. Family history, familial hypercholesterolemia or other lipid metabolism disorders, gender, environmental risk factors and other etiological factors, have been associated with early-onset CAD [5,11]. Lately, many studies have focused on the relationship between inflammatory hematological parameters and coronary artery disease, but there are only limited data regarding the implications of these parameters in young patients with CAD. Based on these data, the current review evaluated the association of inflammatory hematological parameters with early-onset CAD, in order to understand the pathophysiology, and also the implications for risk assessment and prediction.

## 2. Methods

In the field of cardiology, this study stands out as innovative. Few studies have assessed the relationship between these inflammatory markers and AMI in elderly patients. Even rarer are studies that delve into their predictive value for young AMI patients. Given the limited data on this subject, we opted for a narrative review instead of a systematic one. Our research draws from the literature published in the past five years, focusing on adult patients from all age groups who have experienced an acute myocardial infarction. We specifically looked at the link between the mentioned inflammatory markers and their prognostic implications. An exhaustive analysis was conducted using the currently available publications in the MEDLINE, PubMed and EMBASE databases.

## 3. Results

We opted to emphasize results from human subjects research published in the last five years, excluding studies on animals. We organized the presentation of our findings based on these inflammatory markers, comparing the results from adult patients to those of younger patients.

### 3.1. Neutrophil/Lymphocyte Ratio (NLR)

Myocardial infarction (MI) is characterized by two primary phases of inflammation: the inflammatory phase and the proliferative phase. Neutrophils are the initial leukocytes to be observed in the vicinity of tissue injury. The activation of these entities results in the generation of significant quantities of inflammatory mediators that play a crucial role in regulating the body’s response to tissue injury. These mediators are involved in processes such as hypoxic damage, the release of proteolytic enzymes and the production of other signaling molecules. Neutrophils at the site of infarction release free radicals, which serve as an injurious pathway for cardiomyocytes. The release of proteo-enzymes facilitates the removal of the infarct and enhances the recruitment of immune cells, specifically M1 macrophages. Neutrophils play a crucial role in both facilitating the recruitment of macrophages to the infarct site and enabling the effective removal of cellular debris. Lymphocytes are essential in the process of myocardial remodeling after inflammation. An illustration of this would be the presence of CD4+ T regulatory cells, which form a distinct subset of lymphocytes involved in immune regulation with anti-inflammatory properties. These cells are primarily produced in the thymus and are characterized by a high concentration of T cells that exhibit specificity towards autoantigens. The recruitment of proangiogenic macrophages and the formation of collateral arteries are dependent on the presence of T cells [1].

Leukocytes are widely recognized for their significant involvement in the progression of atherosclerosis. This is primarily attributed to their activation, which is linked to compromised rheological properties, a heightened expression of adhesion molecules and the excessive production of cytokines, proteolytic enzymes and reactive oxygen species (ROS). There is a positive correlation between an increased neutrophil count and a heightened risk of ischemic events, as well as mortality associated with acute myocardial infarction (AMI) [2]. The inflammatory condition has an impact on the rheology of neutrophils, resulting in increased cellular rigidity. This rigidity can potentially lead to the obstruction of smaller blood vessels and the initiation of ischemic events [3]. Despite variations in the characterization of high neutrophil-to-lymphocyte ratios (NLRs) across different sample populations, the collective body of research consistently demonstrates a significant correlation between elevated NLR and unfavorable prognostic outcomes among patients with AMI.

Nunez et al. [4] observed that individuals diagnosed with ST-elevation myocardial infarction (STEMI) exhibited the highest level of NLR between 12 and 24 h following admission to the hospital. Furthermore, a higher NLR admission is an independent predictor of increased 30-day all-cause mortality and MACE, and it could be used as a key tool for short-term prognostic assessment in patients with cardiogenic shock that complicates an AMI. Left ventricular thrombosis is another potentially life-threatening complication in post-AMI patients. Previous research has shown that inflammation may play a role in thrombus development; however, it is unclear how inflammation affects thrombus resolution. Patients with post-AMI, who are not having PCI and who have LVT, may have a stronger inflammatory response, as shown by increased NLR and PLR. Therefore, despite anticoagulation, these individuals had reduced LVT resolution. Both NLR and PLR have the potential to isolate a subgroup of LVT patients who need longer-term anticoagulation. Several studies have demonstrated a correlation between an elevated NLR and a greater incidence of no-reflow following percutaneous coronary intervention (PCI) in patients with ST-segment elevation myocardial infarction (STEMI). In the same line, the long-term follow-up of non-ST elevation myocardial infarction (NSTEMI) patients who exhibited slow coronary flow in angiography, a high NLR (NLR > 3.88) was independently related with more frequent recurrent myocardial infarction. In patients diagnosed with NSTEMI, an elevated NLR was found to be correlated with a higher incidence of atrial fibrillation and heart failure, a reduced left ventricular ejection fraction and increased rates of coronary artery bypass grafting (CABG). Others have shown that NLR following hospitalization is a significant and autonomous indicator of both one-year reinfarction and mortality rates among individuals with AMI who also have diabetes [5,6,7,8,9]. NLR was shown to be a prognostic factor for hospitalization and long-term prognosis in patients with STEMI receiving PCI in two meta-analyses that included more than 10,000 patients each [10,11].

Moreover, there was a significant correlation between NLR and myocardial damage, as indicated by elevated levels of CK-MB. Additionally, NLR exhibited a negative correlation with cardiac contractility. So, a high NLR is a robust indicator of the severity of myocardial damage, indicating that the degree of inflammation is linked to impaired contractile function in the heart [1].

A cohort study involving 2618 hospitalized Chinese patients diagnosed with AMI revealed a significant positive correlation between a high NLR exceeding 5.509 and the likelihood of in-hospital mortality among AMI patients. Additionally, another smaller study corroborated these findings by demonstrating that an NLR served as a significant predictor for major adverse cardiovascular events (MACE) in AMI patients [12,13]. In a more recent study, the term “high NLR” was defined as a value greater than 6.69. The researchers focused on older patients with AMI and found that those with high NLR had a 3.091-fold increased risk of in-hospital mortality compared to those with low NLR (95% CI 2.097–4.557, *p* < 0.001). These findings align with previous research that has demonstrated a correlation between a high platelet-to-lymphocyte ratio (PLR) and the risk of in-hospital death in elderly patients with AMI [14,15,16]. Consequently, a high NLR ratio serves as a robust and independent indicator for predicting in-hospital cardiovascular mortality in patients with AMI accompanied by ST-segment elevation [17].

In a retrospective study involving a cohort of over 2500 patients diagnosed with acute coronary syndrome (ACS), NLR demonstrated superior predictive capability for in-hospital mortality compared to other commonly used blood routine examination ratios, such as the neutrophil-to-monocyte ratio (NMR), PLR and lymphocyte-to-monocyte ratio (LMR). Furthermore, NLR exhibited the highest performance in predicting cardiac death among patients with NSTEMI as opposed to those with STEMI. The optimal threshold for NLR in this context was determined to be 5.509. The relationship between NLR and the severity AMI was also observed in accordance with the stratification of the Global Registry of Acute Coronary Events (GRACE) score. Hence, it is plausible to consider that an NLR value greater than 5.509 could serve as a potential indicator for an unfavorable prognosis in patients diagnosed with NSTEMI [13].

In the univariate or multivariate COX analysis, the associations of NLR and MACE (all-cause death, non-fatal ischemic stroke and non-fatal MI) remained significant [18]. There is evidence to suggest that NLR is significantly correlated with the presence of coronary artery calcification (CAC), thereby elevating the risk of developing coronary artery disease (CAD). It is important to point out that patients with ACS who have high NLR levels still do not achieve enough platelet inhibition, even when they receive dual antiplatelet therapy. This inadequate inhibition contributes to thrombosis and raises the likelihood of recurring ischemic events [19,20]. In their study, Tahto et al. demonstrated that the mean values of NLR were significantly elevated in patients diagnosed with AMI compared to those diagnosed with unstable angina. This finding demonstrates the significance of NLR as an inflammatory marker in distinguishing between different clinical presentations of ACS. In the research conducted by Hong et al., a total of 309 patients diagnosed with AMI were examined. These patients underwent cardiac magnetic resonance (CMR) imaging and complete blood cell counts both before and after PCI. The findings of the study revealed that patients with a high post-PCI NLR ratio exhibited an elevated risk of experiencing large-sized infarctions, as determined by CMR imaging. Additionally, these patients were also more likely to experience adverse clinical outcomes. These results highlight the significant prognostic value of post-PCI NLR in individuals diagnosed with AMI [21,22]. After undergoing PCI, NLR and PLR exhibited an initial increase, followed by a subsequent decrease within a 30-day period post-PCI. The Cox multivariate regression analysis revealed that the NLR and PLR measured at 24 h after PCI and STEMI were identified as independent factors that influence the occurrence of MACE following PCI. The analysis of the receiver operating characteristic (ROC) curve indicated that the NLR measured at 24 h after PCI demonstrated superior predictive ability for the occurrence of MACE. There was a significant correlation observed between the NLR at 24 h after PCI and the quantity and length of stents implanted, as well as the duration of the procedure [23].

The use of admission NLR as a cost-effective indicator of inflammation has the potential to assist in the assessment of risk and prognosis for patients diagnosed with LMD/3VD. In a multivariate analysis that accounted for various risk factors, it was found that an NLR exceeding 3.39 independently predicted a higher likelihood of all-cause mortality within a two-year period [24].

In a prospective cohort study conducted in South Korea, notable disparities in mortality rates following STEMI were observed among four distinct groups categorized based on the combination of NLR levels (high, ≥4; low, <4) and the presence of anemia upon admission. These findings were consistent across all patients, including those belonging to various risk subgroups, including low-risk populations. The presence of both NLR and anemia upon admission exhibited a robust correlation with overall mortality following STEMI, even within various low-risk subpopulations [25]. In similar lines, a prior investigation conducted by KAMIR-NIH examined the prognostic significance of the amalgamation of NLR and anemia on the clinical outcomes observed over a span of 180 days in a cohort of 6157 individuals diagnosed with NSTEMI. The study revealed notable variations in clinical outcomes over a 180-day period among three groups categorized based on the NLR and the presence of anemia. The study revealed that the presence of both high NLR and anemia was independently linked to a higher occurrence of major adverse cardiac events within 180 days. This association was observed when comparing it to a reference group with low NLR and no anemia [26].

In patients with NSTEMI, a correlation between presenting NLR and SYNTAX score was shown to be positive. After adjusting for age, sex, and hypertension as confounders, greater NLR at presentation was a reliable predictor of higher SYNTAX score. The SYNTAX score, an angiographic risk stratification score, is used to quantify the degree of coronary involvement and select the most suitable revascularization strategy based on the clinical context in each patient. NLR was not an independent predictor of the SYNTAX score when the Thrombolysis in Myocardial Infarction (TIMI) score was adjusted [27]. In patients with STEMI or NSTEMI treated with PCI, a study conducted to demonstrate the correlation between SYNTAX score (SXs) and NLR, and its association with 1-year cardiovascular (CV) mortality, revealed that an NLR (3.9 and 2.7 for STEMI and NTEMI patients, respectively) was an independent significant predictor of poor prognosis [28]. These studies primarily focused on cohorts comprised predominantly of elderly patients, with limited investigation into younger individuals. In relation to coronary no-reflow and death outcomes, elderly STEMI patients seem to have an initial pro-inflammatory profile that is greater than that of young patients. These findings imply that old and young STEMI patients should receive a distinct therapy strategy. A cut-off point at 65 years old was used to split 625 consecutive STEMI patients receiving primary PCI (pPCI) into two groups for retrospective research. The mean levels of fibrinogen, brain natriuretic peptide (BNP), leukocytes, NLR and C reactive protein/albumin ratio (CAR) were greater in aged patients compared to younger individuals. In contrast, youthful patients had greater albumin levels and lymphocyte counts. Only the BNP value was related with no-reflow in young patients, while the values of NLR, CAR, leukocytes, fibrinogen and neutrophils were associated with no-reflow in older patients. Only BNP and NLR emerged as distinct predictors of all-cause death in the general population and in older individuals after multivariate Cox regression analysis [29]. The occurrence of AMI in young age patients is generally regarded as a relatively uncommon occurrence within the field of medicine. However, it still represents a notable proportion of all AMI cases, ranging from 2% to 10%, as evidenced by various surveys conducted. Contrary to previous studies involving elderly patients, a retrospective cohort study of young individuals with AMI found that NLR did not exhibit prognostic significance in predicting future mortality or serving as an early marker for risk stratification. Nevertheless, when assessed alongside other factors such as hemorheological determinants, plasma markers of platelet and neutrophil activation, oxidative stress, elastase and protein oxidation, it was found to be a valuable indicator [2]. In the same line, two parameters, NLR and plasma viscosity (PV), which were examined concurrently in a prospective study which included young AMI subjects at 3 and 12 months following AMI, exhibited distinct patterns, indicating a substantial decrease in NLR during the follow-up period, while PV did not exhibit a similar change, being the only variable that demonstrated discriminant ability in relation to the cardiovascular complications observed over an 18-month follow-up [6]. NLR is subject to the influence of both diet and physical exercise. Notably, a study involving overweight adolescents demonstrated that a four-week intervention consisting of dietary modifications and physical exercise resulted in a noteworthy decrease in NLR levels, holding significance in light of the extended life expectancy observed in these young individuals with AMI [2].

### 3.2. Platelet/Lymphocyte Ratio (PLR)

Leukocytes and platelets are key players in the pathophysiology of ACS. Following the rupture of atherosclerotic plaques, the activation of platelets is a critical element in the development of coronary thrombosis and occlusion. The development of poor outcomes in people with AMI has been linked to an elevated platelet count, which has been shown to be associated with increased levels of inflammation and platelet activation. However, in the inflammatory and atherosclerotic processes, lymphocytes take a protective function. PLR, a measure created by dividing platelet counts by lymphocyte numbers, has been known as a predictive biomarker for inflammation and negative outcomes in many cardiovascular disorders. Being a biomarker that demonstrates both affordability and widespread accessibility, PLR measurement encompasses both the inflammatory and thrombotic pathways and may provide greater prognostic value than individual platelet or lymphocyte counts. As such, it is a promising candidate for ACS prediction [30,31,32].

Patients with AMI who had successful pPCI within 12 h of the beginning of their chest pain were divided into two groups based on their PLR: low PLR and high PLR, in a prospective longitudinal study and those in the high PLR group had MACE at a rate that was almost 2.8 times higher than that of those in the low PLR group [33]. In patients with ACS following PCI, the PLR demonstrated predictive value and was an independent risk factor for a poor outcome. In comparison to groups without MACE, the PLR was greater in the MACE groups [34]. It was shown that the predictive utility of PLR is restricted in patients with ACS in research to evaluate the prognostic importance of five lymphocyte-based inflammatory markers. Therefore, for the aim of risk stratification, it would be important to take into account combining PLR with other indices. In comparison to either PLR or NLR alone, Liu et al.’s study showed that PLR and NLR together had improved predictive capacities for AMI. The PLR–NLR combination also shown increased sensitivity in predicting the outcome of AMI [18,35,36]. The PLR may be more useful than platelet or lymphocyte counts alone in determining prognosis since it incorporates the predictive qualities of both counts. Previous research has shown that PLR is important for predicting outcomes in people with ACS. This suggests that PLR could potentially serve as a reliable prognostic biomarker in ACS patients, aiding in the identification of risk levels and guiding appropriate therapeutic interventions [31,37]. In addition, a retrospective study that separated the population into two groups based on the PLR cutoff value found that high PLR was linked to a higher risk of short-term death (28 days) in severely sick NSTEMI patients [38]. In contrast, it was recently shown that, although higher levels of NLR and PLR were linked to poorer outcomes in ACS patients, they did not serve as independent predictors of MACE. The systemic immune-inflammation index (SII), which is based on neutrophils, lymphocytes, and platelets, was shown to accurately predict both short- and long-term MACE in ACS patients [39].

In patients with AMI undergoing pPCI, multicenter retrospective cohort research found a link between severe heart failure and in-hospital MACEs and high PLR as independent risk factors for inadequate myocardial reperfusion. Furthermore, the average diameter of the implanted stent was substantially lower, thrombus aspiration was greater and platelet count was higher in the high-PLR group. Additionally, following PCI, the TIMI grade and MBG stage in those individuals were much lower [40]. In STEMI, the occurrence of coronary no-reflow is linked to a poor clinical prognosis. PLR was considerably greater in the no-reflow group compared to the group with normal reflow in a total of 200 patients with STEMI who had pPCI. No-reflow was detected in 29% of these patients [41]. Similar to this, Enöz O. et al. claim that PLR is a widely accessible inflammatory biomarker that may be used to predict the no-reflow event after thrombus aspiration during PCI in patients with STEMI [42]. With reference to the correlation between intracoronary thrombus load and SII, NLR and PLR in STEMI patients who had pPCI, high values of these parameters were discovered to be independent predictors of high thrombus burden. However, compared to NLR and PLR, SII’s predictiveness was greater [43]. In a study that examined blood markers of inflammation in people with ACS and a history of CABG, it was determined that those who received unsuccessful SVG PCI had a more severe inflammatory condition than those who received successful saphenous vein graft (SVG) PCI, and PLR is an important and independent indicator of ineffective SVG [44].

When examined based on the HEART score (a clinical scoring system for patients presenting with chest pain at the emergency department and used in diagnostic and therapeutic decisions), PLR and NLR proved to be useful instruments for detecting high-risk individuals in a cross-sectional study of 120 patients who attended the emergency department with acute chest pain [45]. In patients with AMI undergoing coronary angiography, a retrospective study that examined the relationship between PLR and in-hospital MACEs and the severity of CAD as measured by the Gensini score (GS) has shown that high PLR plays a significant role as an independent risk predictor of in-hospital MACEs and CAD severity. This implies that PLR may be employed as a predictive sign for the GS’s evaluation of the severity of high-risk AMI and CAD [46]. In another meta-analysis, Li et al. found that higher PLR levels were independently associated with all-cause mortality and cardiovascular events in patients with ACS, with a 2.24-fold increased risk of short-term adverse outcomes and a 2.32-fold increased risk of long-term adverse outcomes for those with higher PLR levels [47]. In addition, the degree of PLR is associated with the occurrence of Left Ventricular Thrombus (LVT), one of the most frequent sequelae of acute anterior myocardial infarction and one that is connected to the potentially fatal consequences of thromboembolism or stroke. Increased PLR and DTBT (time from emergency department evaluation (“door” time) to coronary balloon inflation) and the presence of LV aneurysm were associated with the presence of LVT and were independent predictors of LVT formation in acute anterior STEMI patients with LV dysfunction [48]. Furthermore, a recent study showed that NLR and PLR were positively linked with carotid atherogenesis in young individuals with newly diagnosed type 2 diabetes [49].

Currently, there is a lack of available research that has examined the relationship between PLR and young individuals with AMI. Issada et al. [50] did show that the link between PLR and CAD is affected by age, however. High PLR was an independent indicator of CAD in older high-risk patients, but it was inversely connected with early CAD in younger patients. According to Kazem et al. [51], the PLR has a significant and distinct age-specific correlation with cardiovascular mortality in ACS patients. The PLR provides only for the identification of patients >65 years old who are at high risk for catastrophic events after ACS—even in the long term—but not in those under the age of 65 years.

### 3.3. Monocyte/Lymphocyte Ratio (MLR) and Lymphocyte/Monocyte Ratio (LMR)

Monocytes serve as an essential part of the innate immune system and play an active role in the processes associated with endogenous inflammation. In response to biological signals, these cells have the ability to migrate from the bloodstream to different tissues and undergo differentiation into various types, including inflammatory dendritic cells, macrophages and foam cells. This process triggers the secretion of proinflammatory cytokines, the production of matrix metalloproteinases and the formation of reactive oxidizing substances. Consequently, this mechanism enables the accumulation of a significant number of inflammatory cells that possess the capability to infiltrate the region impacted by infarction, thereby exacerbating the damage inflicted upon myocardial cells in the ischemic area. Ultimately, this process leads to the degradation and breakdown of the myocardial cell membrane, the fibrin cytoskeleton and, finally, ends in the death of the cell [52]. The study conducted by Fan et al. [53] revealed a noteworthy correlation between a heightened monocyte-to-lymphocyte ratio (MLR) and an augmented mortality risk during the 6-month post-intervention period in patients diagnosed with AMI who underwent PCI.

Five lymphocyte-based inflammatory indices (PLR, NLR, MLR, SII, and SIRI) were evaluated to determine their prognostic value in predicting long-term outcomes and to improve the prognostic value of the GRACE risk score for the classification of risk of patients with ACS who underwent PCI. The study’s findings showed a substantial and independent relationship between these indices and MACE [18]. Additionally, in a total of 523 patients with AMI who were above the age of 80, a recent prospective study examined the predictive significance of leukocyte markers for cardiovascular death. In extremely elderly individuals, leukocyte subtypes were independently correlated with cardiovascular mortality, according to Cox regression models. However, the best leukocyte markers for predicting death were neutrophils-plus-monocytes-to-lymphocytes ratio (NMLR) and NLR [54].

One of the most dangerous side effects of AMI is myocardial rupture. In two distinct retrospective investigations, Dai et al. demonstrated that a high MLR, together with neutrophil percentage-to-albumin ratio (NPAR) and monocyte-to-hematocrit ratio, were independent risk factors for cardiac rupture and free-wall rupture, respectively [52,55]. According to a study using optical coherence tomography (OCT) to examine coronary non-culprit plaque in terms of the susceptibility in patients with ACS, non-culprit plaque was more vulnerable in ACS patients with high MLR and had thinner fibrous cap thickness, a larger maximum lipid angle and an extended lipid bacteria dimension. Meanwhile, the high MLR group had a higher rate of plaque rupture and thin cap fibro-atheroma (TCFA) observed by OCT. Additionally, it has been shown that a high MLR level is an individual risk factor for TCFA [56]. Higher admission NMLR and older age were shown to be independent risk factors for in-hospital death following a multivariate Cox regression analysis of current data [57].

In addition to MLR, LMR has also been evaluated as a marker for prognosis in ACS patients and a tool for determining MI risk stratification. In conclusion, LMR was shown to be a reliable predictor of in-hospital, long-term mortality [58,59] and 1-year mortality in critically ill patients with AMI [60]. There is currently no established correlation when it comes to younger patients with AMI.

### 3.4. Monocyte/High-Density Lipoprotein Cholesterol Ratio

Circulating monocytes play a crucial role in the development of atherosclerotic plaques, while high-density lipoprotein cholesterol (HDL-C) molecules effectively suppress their functions. HDL-C molecules hinder the differentiation of monocytes into macrophages, impede their migration, facilitate the removal of cholesterol from these cells and additionally inhibit the oxidation of LDL-C [61,62,63]. In patients with ACS, recent research found a correlation between the monocyte-to-HDL-C ratio (MHR) and an enhanced risk of MACE as well as death. According to this study, MHR may be used as a prognostic biomarker for ACS [64,65]. In a prospective study, a total of 2661 consecutive ACS patients were recruited and monitored for an average of 31.6 months. Multivariate logistic regression analysis revealed that patients in the third MHR tertial group had hazard ratios of 1.303 for long-term MACE and 1.406 for in-hospital MACE [66].

The chance of Iing a higher mortality rate during hospitaIization is positively correlated with the heart rate of STEMI patients who have received pPCI. MHR proved to be a novel predictor and may be frequently utilized as a biomarker in the assessment of the risk and severity of atherosclerosis in AMI. It predicted the risk and severity of atherosclerosis in these categories of patients [61]. Furthermore, research found that, among CAD patients in China who had PCI, an elevated MHR was independently related with long-term mortality. The largest mortality risk was seen in CAD patients who received PCI and fell into the highest MHR quartile [67]. According to Sercelik et al. [68], the MHR was considerably greater in the STEMI group compared to the control group and in the high TIMI score group compared to the low TIMI score group. In a recent study, Karim et al. looked at the correlation between the TIMI score, MHR and GRACE in patients with ACS. The results showed that the MHR values was considerably greater in the group with an increased TIMI score. The MHR was also more significant in the high-risk GRACE category than in the low-risk GRACE cohort [69].

Aiming to determine the predictive value of MHR at the time of hospital admission in late cardiac remodeling and subsequent 1-year mortality admission, prospective multicenter research focused on patients with acute ST-elevation MI. Patients who had unfavorable cardiac remodeling (AR) had higher MHR values. In those with and without AR, a positive connection between MHR and infarct size was found. It was also shown that MHR has a strong diagnostic ability for indicating AR and is an independent predictor of AR. A 1% rise in MHR raised the chance of AR by 3.21 times, while patients with MHR of >1.6 had a 5.62 times greater mortality risk [70].

Reduced LMR and greater MHR were found to be independent predictors of the slow flow/no-reflow phenomenon in a retrospective study that included 426 NSTEMI patients divided into non-slow flow/no reflow and slow flow/no reflow groups based on postintervention thrombolysis in myocardial infarction flow grade [71]. On the same note, there is currently no established correlation when it comes to younger patients with AMI.

**Figure 1:**
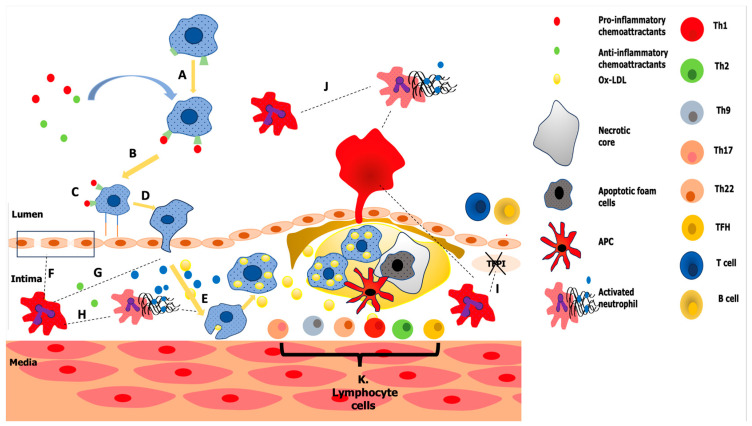
The role of monocytes, neutrophils and lymphocytes in atherosclerotic plaque formation. A, B: Activated endothelial cells produce chemoattractants and adhesion molecules that draw in monocytes. The binding of inflammatory chemoattractants by monocytes from the blood promotes their chemotaxis to the atherosclerotic endothelium; C, D: Inflammatory monocytes then move intima by adhering to the atherosclerotic endothelium; E: Once reaching the location of the lesion, monocytes differentiate into macrophages, which further contribute to inflammation. Ingesting cholesterol causes these cell types to develop into foam cells, thus contributing to the formation of atherosclerotic plaque. Although these macrophages initially help by removing these lipoproteins from the subendothelium, with time the macrophages become engorged with lipids, which leads to dysregulated lipid metabolism. These foam cells eventually experience apoptosis and necrosis, and if they are not removed effectively by M2 macrophages through efferocytosis, they will release their toxic and pro-inflammatory contents into the subendothelial space. This will further encourage cell death and inflammation, the development of the necrotic core and further increase the vulnerability of the plaque. The plaque is susceptible to rupture when the fibrous cover thins and the necrotic core expands, which might cause a thrombosis or other acute cardiovascular event. F: Endothelial cell lesions result from the breakdown of the basement membrane by neutrophilic MMP, MPO and ROS. G: Neutrophils produce chemoattractant for monocytes. H: NETs complexes trigger macrophages to release the proinflammatory cytokine. NETosis, a cell death mechanism distinct from apoptosis or necrosis, is the method by which these chromatin complexes are released from neutrophil nuclei. NETosis enables neutrophils to destroy infections more effectively by creating a mechanical barrier to “trap” germs. I: Neutrophils block the tissue factor plasminogen inhibitor, which causes thrombus to develop. J: Neutrophils that have been activated produce NETs complexes, which promote thrombus development. K: T lymphocyte responses are induced by APCs such as macrophages, DCs and B cells. There are many subsets of naive CD4+ T helper (Th) cells, and stimulatory chemicals in fact encourage T cells to express transcription factors that result in differentiation into Th phenotypes. Both pro-atherogenic and atheroprotective properties may be seen in CD4+ T cells. Th1, Th2, Th9, Th17, Th22, Treg and follicular helper T (TFH) cells may be detected in atherosclerotic lesions. By releasing IFN-γ, interleukins, TGF-β and protease, these lymphocytes promote inflammation, endothelial cell death, plaque cell apoptosis, monocytic migration, and thus the development of atherosclerotic plaques, foam cells and atherosclerotic plaque rupture. Type I NKT cells may stimulate immune cells in plaque by secreting cytokines and accelerating the development of atherosclerosis. The cytotoxic effects of CD8+ T cells, in contrast with lesion-stabilizing cells and the generation of inflammatory cytokines by CD8+ T cells, may worsen the growth and instability of lesions by escalating inflammatory reactions in plaques of atherosclerosis. L. White blood cells of the lymphocyte subtype known as B cells, or B lymphocytes, are classified into two separate groups (B1 and B2). By secreting antibodies, B cells are essential to the humoral immunity part of the adaptive immune system. B cells also present antigens, which are classified as expert APCs, and release cytokines. B lymphocytes, which aid in the development of lymphoid follicles, may contribute to atherosclerosis. At all phases of the disease’s development, IgG and IgM antibodies may be found in atherosclerotic plaques. Ox-LDL—oxidized low-density lipoprotein; TFPI—tissue factor plasminogen inhibitor; MMP—matrix metalloproteinases; MPO—myeloperoxidase; ROS—reactive oxygen species; NETs—neutrophil extracellular traps; APCs—antigen-presenting cell; DCs—dendritic cells; IFN-γ—interferon gamma; TGF-β—transforming growth factor beta; NKT—natural killer T; IgG—immunoglobulin G; IgM—immunoglobulin M. There are some potential factors that may influence the inflammatory hematological ratios.

Thus, atherosclerosis and inflammation are pivotal in the development of CAD, with the connection emphasized by the involvement of immune cells like neutrophils, lymphocytes, monocytes and macrophages (Figure 1) [72,73,74,75,76].

Thus, neutrophilia can be broadly categorized into primary and secondary forms. Primary neutrophilia has its origins in conditions intrinsic to the bone marrow. The causes of primary neutrophilia encompass chronic idiopathic leukocytosis, myeloproliferative disorders like chronic myeloid leukemia, essential thrombocythemia, polycythemia vera and chronic myelomonocytic leukemia. There are also genetic or inherited conditions that result in primary neutrophilia, such as the transient myeloproliferative disorder seen in Down syndrome and the rare condition of hereditary chronic neutrophilia. Secondary neutrophilia, on the other hand, is typically a response to external stimuli or conditions. Infections and inflammation are leading contributors. An important manifestation to highlight is the leukemoid reaction, which can be seen in diseases like Clostridium difficile infection or tuberculosis. Both acute and chronic inflammatory conditions can lead to neutrophilia, with examples ranging from rheumatoid arthritis (in both its juvenile and adult forms), granulomatous diseases, vasculitis, myositis, chronic hepatitis, to inflammatory bowel diseases. Solid tumors can also give rise to neutrophilia, and this can be due to reasons such as a paraneoplastic leukemoid reaction, bone marrow involvement by the tumor, nonspecific inflammation related to the tumor, concurrent infections, or the use of high-dose corticosteroids in the treatment of the tumor. Furthermore, various drugs can induce neutrophilia. These include glucocorticoids, lithium, catecholamines, as well as recombinant granulocyte and granulocyte-macrophage colony-stimulating factors, each acting through different mechanisms. Lastly, there is what is termed “factitious neutrophilia.” This is not a true increase in neutrophils but rather an artifact seen due to various factors during the blood sample’s laboratory processing [77].

Lymphopenia, also known as lymphocytopenia, refers to a reduced lymphocyte count in the blood, typically falling below 1000/mcL (1 × 10^9^/L) in adults. The origins of lymphopenia can be both acquired and congenital. Acquired causes of lymphopenia span various conditions. Infectious agents such as HIV, viral hepatitis, influenza, SARS-CoV-2, tuberculosis and certain types of pneumonia can lead to a decreased lymphocyte count. Autoimmune disorders, including Sjögren’s syndrome, lupus and rheumatoid arthritis, have been linked to lymphopenia. Hematological malignancies and disorders like Hodgkin’s disease and aplastic anemia can also result in reduced lymphocytes. Medical interventions and conditions, such as blood or bone marrow transplants, chemotherapy or radiation for cancer, steroid therapy, major surgeries, excessive alcohol consumption and malnutrition, can also induce lymphopenia. On the inherited front, certain genetic disorders are associated with lymphopenia. These include Ataxia telangiectasia, chromosome 22q11.2 deletion syndrome, common variable immunodeficiency, severe combined immunodeficiency syndrome and Wiskott–Aldrich syndrome. Each of these genetic conditions can manifest with a decreased number of lymphocytes as part of their clinical picture [78,79,80].

Thrombocytosis refers to a platelet count that exceeds the upper limit of the normal reference range, typically beyond 350,000 to 400,000/mL. This condition can be classified into two primary types: primary (or clonal) thrombocytosis and secondary (or reactive) thrombocytosis. Reactive thrombocytosis can be triggered by a variety of factors. These include iron deficiency, infections, rheumatological conditions, inflammation, recent surgical procedures or trauma, malignancies, absence of spleen function (hyposplenism) or surgical removal of the spleen (splenectomy), rapid blood loss, medications and even certain myeloproliferative disorders. Primary or clonal thrombocytosis, on the other hand, is typically associated with an underlying myeloproliferative or myelodysplastic neoplasm. Recently, there has been an increased recognition of acquired “driver” mutations that promote uncontrolled cell growth, owing to aberrantly activated cellular signaling pathways. These mutations lead to the autonomous proliferation of the affected cells, contributing to the elevated platelet counts seen in primary thrombocytosis [81].

Monocytosis in adults is typically characterized by absolute monocyte counts ranging from 0.2 to 0.8 × 10^9^/L. These values can vary significantly depending on age and gender. Broadly, monocytosis can be grouped into two primary categories based on its causes:

- Reactive monocytosis: This form arises as a response to various conditions such as acute infections, post-splenectomy status, chronic infections and rheumatologic diseases.
- Clonal monocytosis: This is associated with hematologic malignancies and disorders like acute monocytic or myelomonocytic leukemia, chronic myelomonocytic leukemia, juvenile myelomonocytic leukemia, essential thrombocythemia, polycythemia vera and primary myelofibrosis [82].

Many research studies exclude specific populations when evaluating hematological parameters. Excluded groups often consist of: patients with inflammatory diseases like gastritis, chronic cholecystitis, nephritis, rhinitis, pharyngitis, bronchitis, and myocarditis; individuals with inflammatory diseases (e.g., gastritis, chronic cholecystitis, nephritis, rhinitis, pharyngitis, bronchitis, myocarditis), conditions such as rheumatoid arthritis and gout; those with immune system disorders; patients who have recently experienced trauma or have had surgery; those who have received a blood transfusion within the past month; individuals diagnosed with blood-related diseases; patients who have been administered steroids or have recently undergone treatments like radiotherapy, chemotherapy, or immunotherapy. By excluding these specific groups, researchers aim to achieve more accurate and representative findings without the interference of confounding variables that could potentially affect the measured values of these hematological parameters.

To improve the clarity of the study, we systematized, in a comparative table, the parameters studied in the elderly and young, with their implications (Table 1).

## 4. Limits

Due to the limited research available on this topic, we undertook a narrative review rather than a systematic one, which is a notable constraint of our study. There is a scarcity in the literature regarding studies focusing on the role of inflammatory hematological markers in young individuals with ischemic myocardial infarction. However, it is crucial to underscore that our study is pioneering and paves the way for subsequent research in this area. Another limitation of our research is the preponderance of single-center and retrospective studies in our analysis, as well as the links between these inflammatory hematological markers and other coexisting medical conditions. Most researchers used a one-time measurement of parameters at the time of admission, rather than repeated measurements. This points to a pressing need for larger, multicenter studies to thoroughly examine this relationship.

## 5. Future Directions

Future research should focus on understanding the specific mechanisms by which acute myocardial infarction in young patients triggers an immediate inflammatory reaction. Insights from these studies can lead to more precise anti-inflammatory treatments and steer the direction of future drug discoveries. Given the vast amount of preclinical and observational evidence pointing to the detrimental impacts of inflammation around the myocardial infarction in adult patients, there is a need for larger randomized trials in young patients. These trials, set to measure concrete clinical outcomes, should assess if treating inflammation can enhance both short-term and long-term results in young patients with acute myocardial infarction.

## 6. Conclusions

The relationship between inflammatory hematological parameters and CAD is undeniable. As such, a deeper exploration into their role in early-onset CAD is crucial for better risk stratification, therapeutic targeting and prognosis evaluation. Comprehensive studies in this realm can potentially pave the way for more personalized and effective treatment modalities for acute myocardial infarction, especially in younger populations.

## Figures and Tables

**Table 1:** Predictive value of inflammatory hematological ratios in acute myocardial infarction and particularities in elderly vs. young patients.

Ratio	Predictive Value in Elderly Patients	Predictive Value in Young Patients
NLR	Higher NLR value at admission: independent predictor of increased in hospital mortality, 30-day all-cause mortality and MACE in AMI patients [4,12,13,17,18]	Limited data NLR have no prognostic significance in predicting future mortality or early risk stratification [6].
	Higher NLR post-AMI value: decreased rate of LVT resolution and the need for longer-term anticoagulation [9] In STEMI, an increased NLR: predictor of hospitalization, an unfavorable long-term prognosis, a higher frequency of no-reflow after PCI and overall mortality [10,11,25,29]. In NSTEMI, an elevated NLR value corelated with: higher incidence of AF and HF, a reduced LVEF, increased rates of CABG, elevated GRACE and SYNTAX scores and higher occurrence of MACE within 180 days [6,7,8,13,26,27]. Higher NLR value: indicator of the severity of myocardial damage and thus an elevated risk of experiencing large-sized infarctions [1,21,22]. The number and length of stents inserted, the duration of the operation, were significantly correlated with the NLR at 24 h following PCI, increasing the risk of recurrent ischemic episodes [19,20,23].	
PLR	High PLR demonstrated predictive value and was an independent risk factor for both short-term and long-term MACE, inadequate myocardial reperfusion in AMI patients [31,34,37,40,47].	Limited data concluded that high PLR is inversely connected with early CAD in younger patients [50,51].
	High PLR was linked to a higher risk of short-term death (28 days) in severely sick NSTEMI patients [38]. High levels were independent predictors of the no-reflow phenomenon and intracoronary thrombus burden [41,42,43]. Is an important and independent indicator of ineffective SVG [44]. High PLR is corelated with an elevated HEART score [45]. Is an independent risk factor for in-hospital MACEs and CAD severity, according to the Gensini score [46]. In anterior STEMI patients with LV dysfunction, this ratio was an independent predictor of LVT development [48].	
MLR	High MLR value together with NPAR and monocyte-to-hematocrit ratio were independent risk factors for cardiac rupture and free-wall rupture [54]. High MLR is an individual risk factor for TCFA and increased the chance of plaque rupture [56]. In patients with AMI who had PCI, there is a correlation between an elevated MLR and an increased death risk during the subsequent six months as well as predicting long-term MACE [18,53].	No correlation regarding young patients with AMI.
LMR	Reliable predictor of in-hospital, long-term mortality and 1-year mortality in critically ill patients with AMI [58,59,60].	No correlation regarding young patients with AMI.
MHR	Higher MHR values corelated with long-term mortality and MACE, in-hospital MACE [66,67]. Predicted the risk and severity of atherosclerosis in AMI patients [67]. In comparison to the low-risk cohort, MHR values were significantly higher in the group with an elevated TIMI score and high-risk GRACE category [68,69]. Is an independent predictor of both AR and infarct size [70]. Higher values were found to be independent predictors of the slow flow/no-reflow phenomenon [71].	No correlation regarding young patients with AMI.

NLR—neutrophil/lymphocyte ratio; PLR—platelet/lymphocyte ratio; MLR—monocyte/lymphocyte ratio; LMR—lymphocyte/monocyte ratio; MHR—monocyte/HDL-cholesterol ratio; AMI—acute myocardial infarction; LV—left ventricle; LVEF—left ventricular ejection fraction; MACE—major adverse cardiac events; CAD—coronary artery disease; NSTEMI—non-ST elevation myocardial infarction; STEMI—ST-segment elevation myocardial infarction; LVT—Left Ventricular Thrombus; PCI—percutaneous coronary intervention; AF—atrial fibrillation; HF—heart failure; CABG—coronary artery bypass grafting; SVG—saphenous vein graft; NPAR—neutrophil percentage-to-albumin ratio; TCFA—thin cap fibro-atheroma; AR—unfavorable cardiac remodeling.
